# Supplementary material for: The impact of the embryonic DNA methylation program on CTCF-mediated genome regulation
Source: Nucleic Acids Res. 2024 Aug 24;52(18):10934–50. doi: 10.1093/nar/gkae724 (PMC11472158; doi:10.1093/nar/gkae724)
Supplement: gkae724_Supplemental_Files [file gkae724_supplemental_files.zip › Supplementary Figures S1-13.pdf]

Supplementary Figure S1

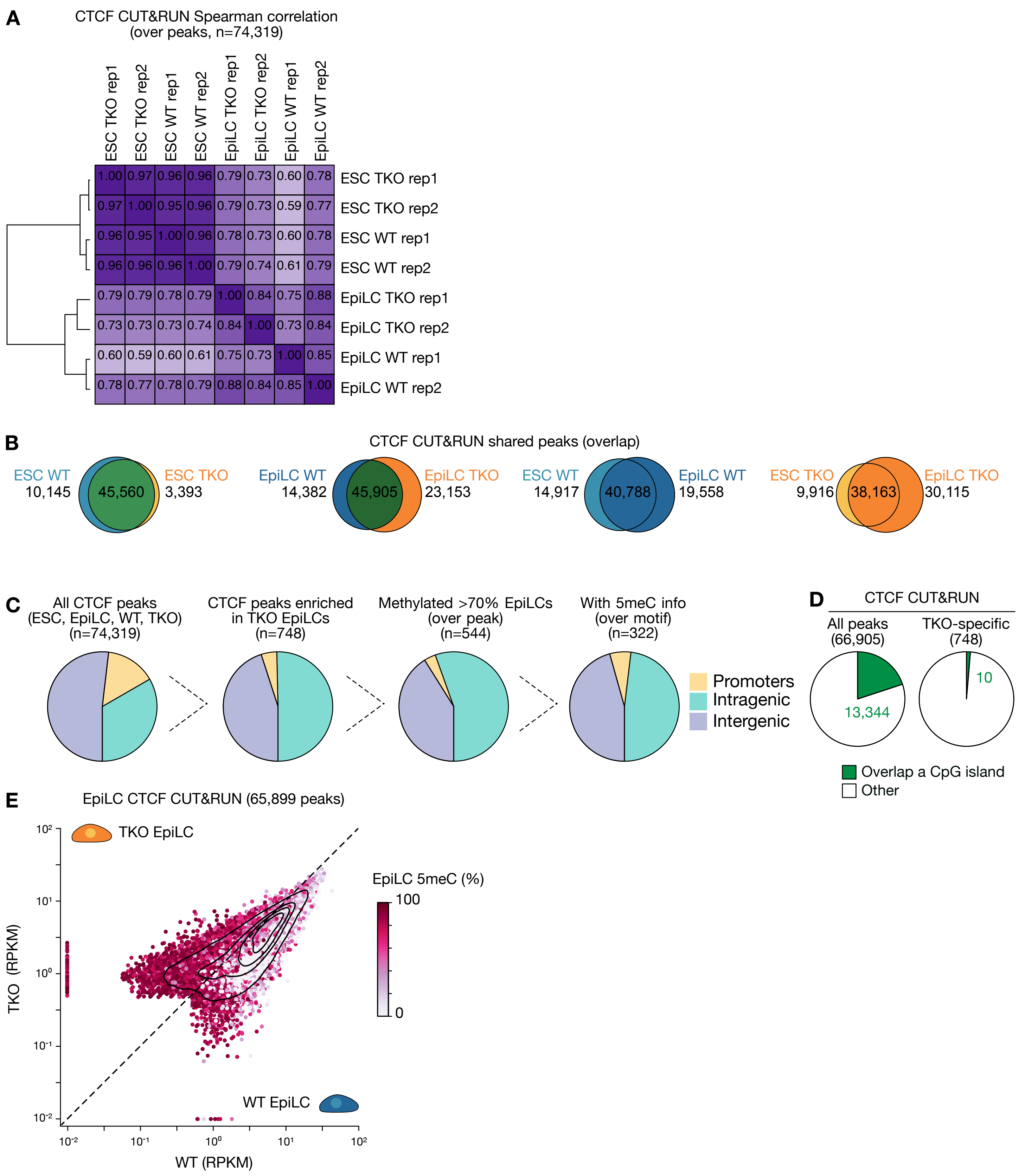

Supplementary Figure S1 – Profiling CTCF occupancy in WT and TKO ESCs and EpiLCs by CUT&RUN.
A. Correlogram showing the Spearman correlation of CTCF enrichment over CTCF peaks in WT and TKO ESCs and EpiLCs. Hierarchical clustering of datasets is included (left).
B. Overlap between CTCF peaks in WT and TKO ESCs and EpiLCs. The number of peaks unique to and overlapping between cells are indicated.
C. Pie charts showing the distribution of CTCF binding sites over promoters, intragenic regions (gene bodies) and intergenic regions from the CUT&RUN data. CTCF binding sites are categorized based on CTCF enrichment in TKO EpiLCs (fold-change>2, adj. pval<0.05), those enriched in TKO EpiLCs that normally gain >70% 5meC in WT EpiLCs, and those for which 5meC levels can be ascertained over their canonical motif.
D. Fraction of CTCF binding sites that overlap a CpG island, categorized by whether they are TKO-specific or not.
E. 2D scatterplot showing CTCF enrichment over CTCF peaks in WT and TKO EpiLCs as in **Figure 1**. Data points are colored based on mean 5meC levels in WT EpiLCs. Only peaks with at least one CpG for which 5meC levels could be assessed (5X read coverage) are shown (n=65,899).

Supplementary Figure S2

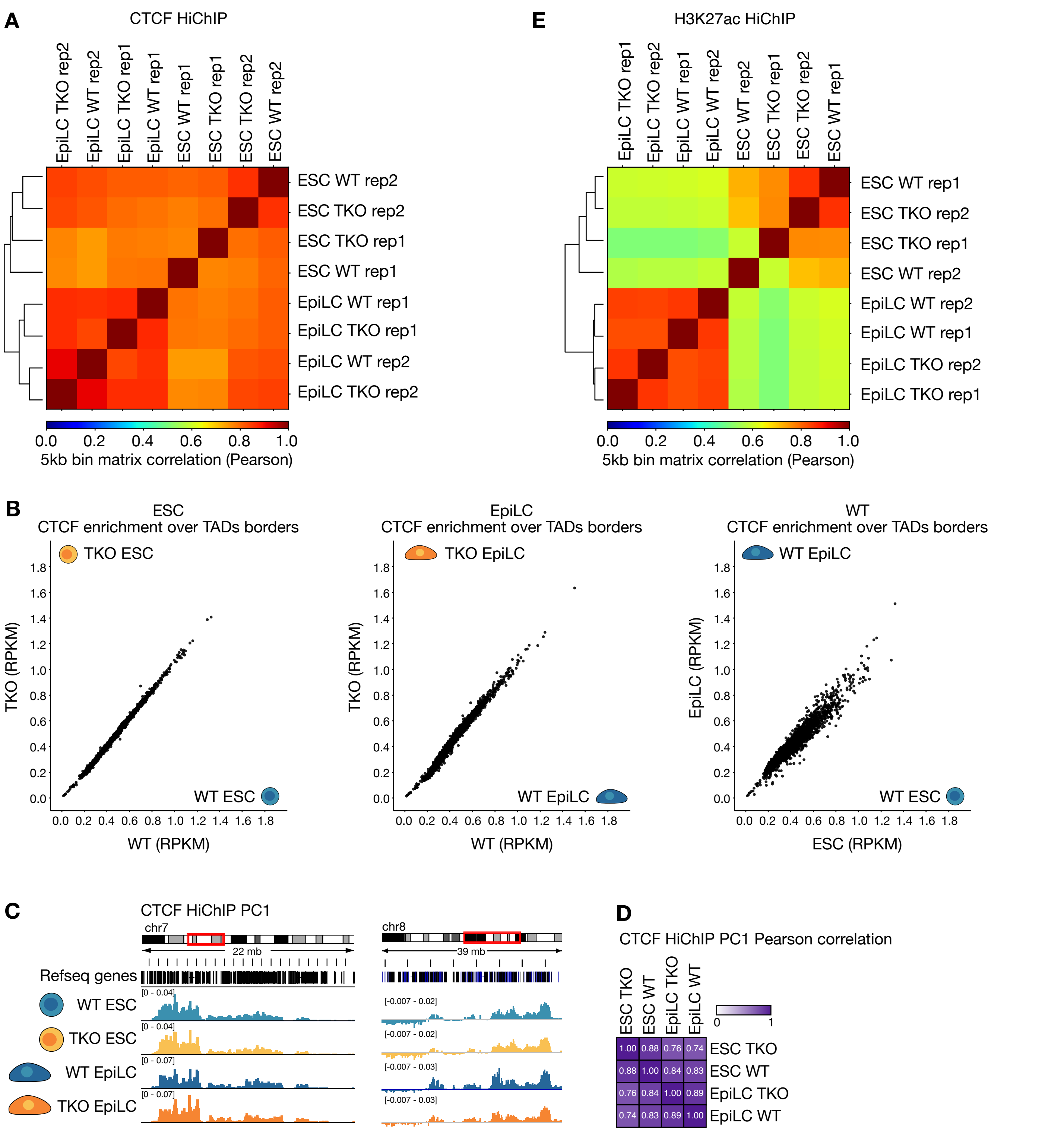

Supplementary Figure S2 – Profiling 3D CTCF interactions in WT and TKO ESCs and EpiLCs.
A. Correlogram depicting the Pearson correlation of 3D contacts over 5kb bins from CTCF HiChIP in WT and TKO ESCs and EpiLCs.
B. 2D scatter plots showing CTCF enrichment (RPKM) over TADs in WT and TKO ESCs and EpiLCs (n=2,426).
C. Integrative Genome Viewer screenshots showing TAD compartmentalization (PC1) in CTCF HiChIP data.
D. Correlogram showing Pearson correlation levels of TAD compartmentalization (PC1) over 100 kb bins using CTCF HiChIP data.
E. Correlogram showing the Pearson correlation of 3D contacts over 5kb bins from H3K27ac HiChIP in WT and TKO ESCs and EpiLCs.

Supplementary Figure S3

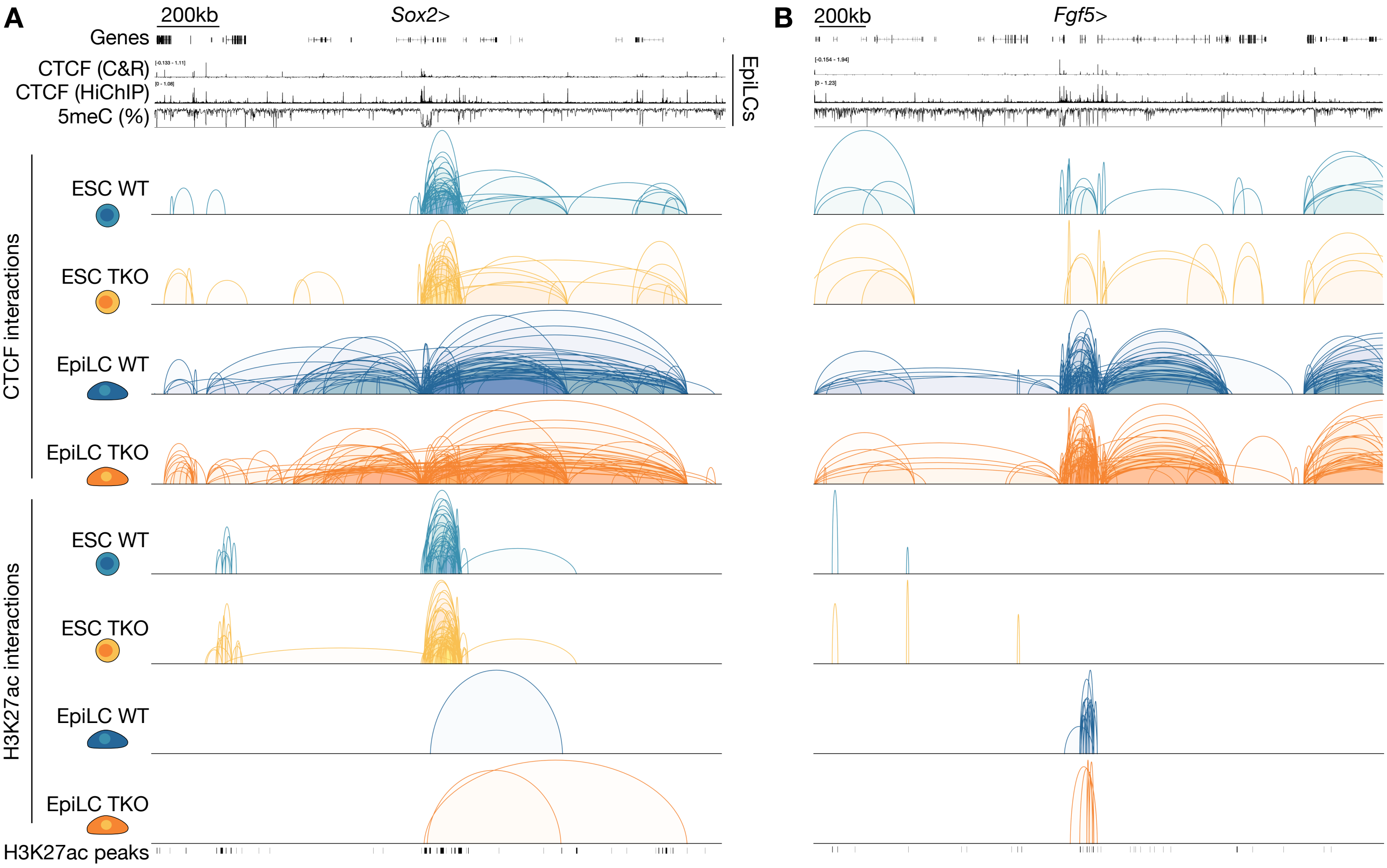

Supplementary Figure S3 – Megabase view of 3D interaction data.
A. Integrative Genome Viewer screenshot of the ESC-marker gene *Sox2* (1.8Mb). Genes, CTCF enrichment (CUT&RUN, HiChIP) and WGBS data from WT EpiLCs are included. Significant 3D interactions (peak-to-peak and peak-to-other) are displayed as loops (FDR<0.05, >4 read coverage). H3K27ac peaks called from HiChIP data are shown. Coordinates: chr3:33,792,031-35,603,556.
B. Screenshot of the EpiLC marker gene *Fgf5*, (2.4Mb) as shown in **A.** Coordinates: chr5:97,126,016-99,537,408.

Supplementary Figure S4

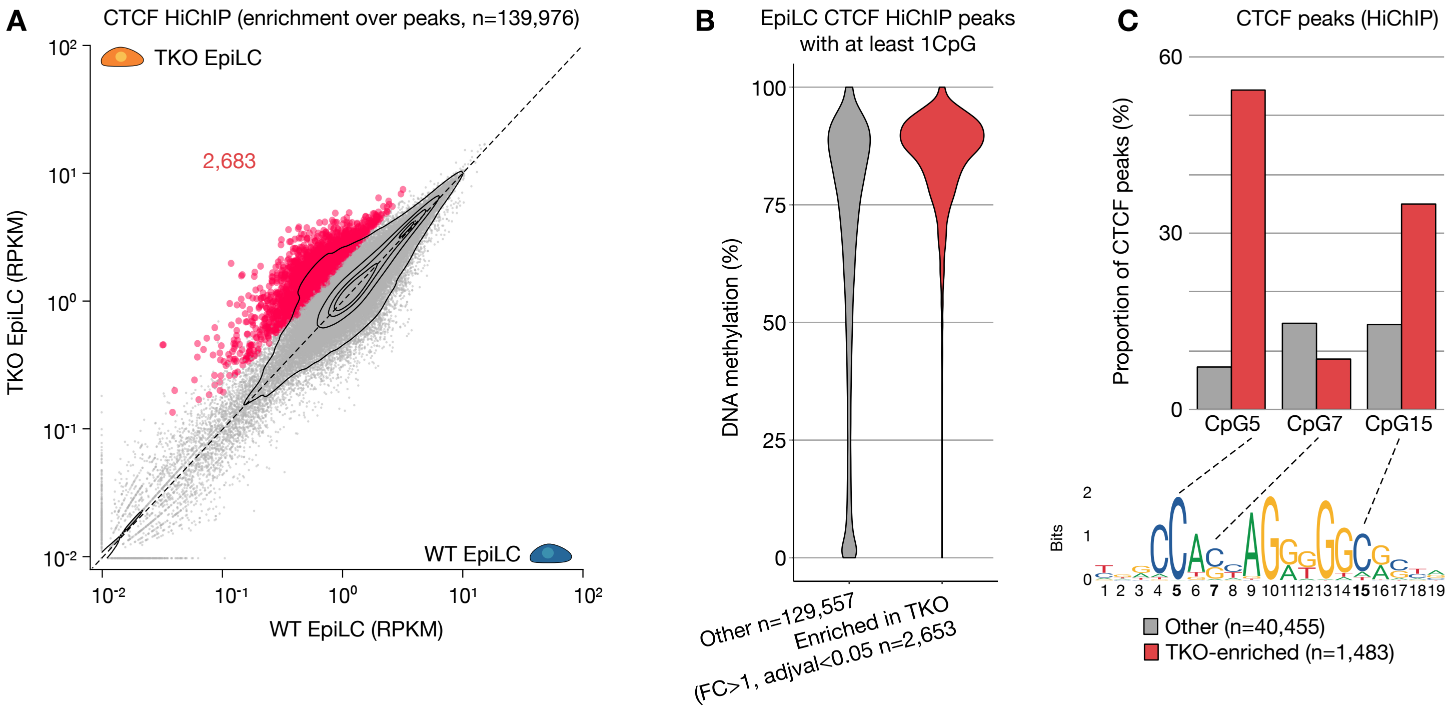

Supplementary Figure S4 – CTCF exhibits potential 5meC sensitivity in CTCF HiChIP data.
A. 2D scatterplot showing CTCF enrichment (RPKM) over CTCF peaks in WT versus TKO EpiLC HiChIP data. CTCF peaks enriched in TKO EpiLCs are highlighted in red.
B. Violin plot of the distribution of EpiLC CpG methylation levels within CTCF peaks. Peaks are categorized as in **A**, and only those for which 5meC levels could be assessed (5X coverage) are included.
C. Bar plot showing the proportion of CTCF peaks that overlap a canonical CTCF binding motif with a CpG at position 5, 7 or 15.

Supplementary Figure S5

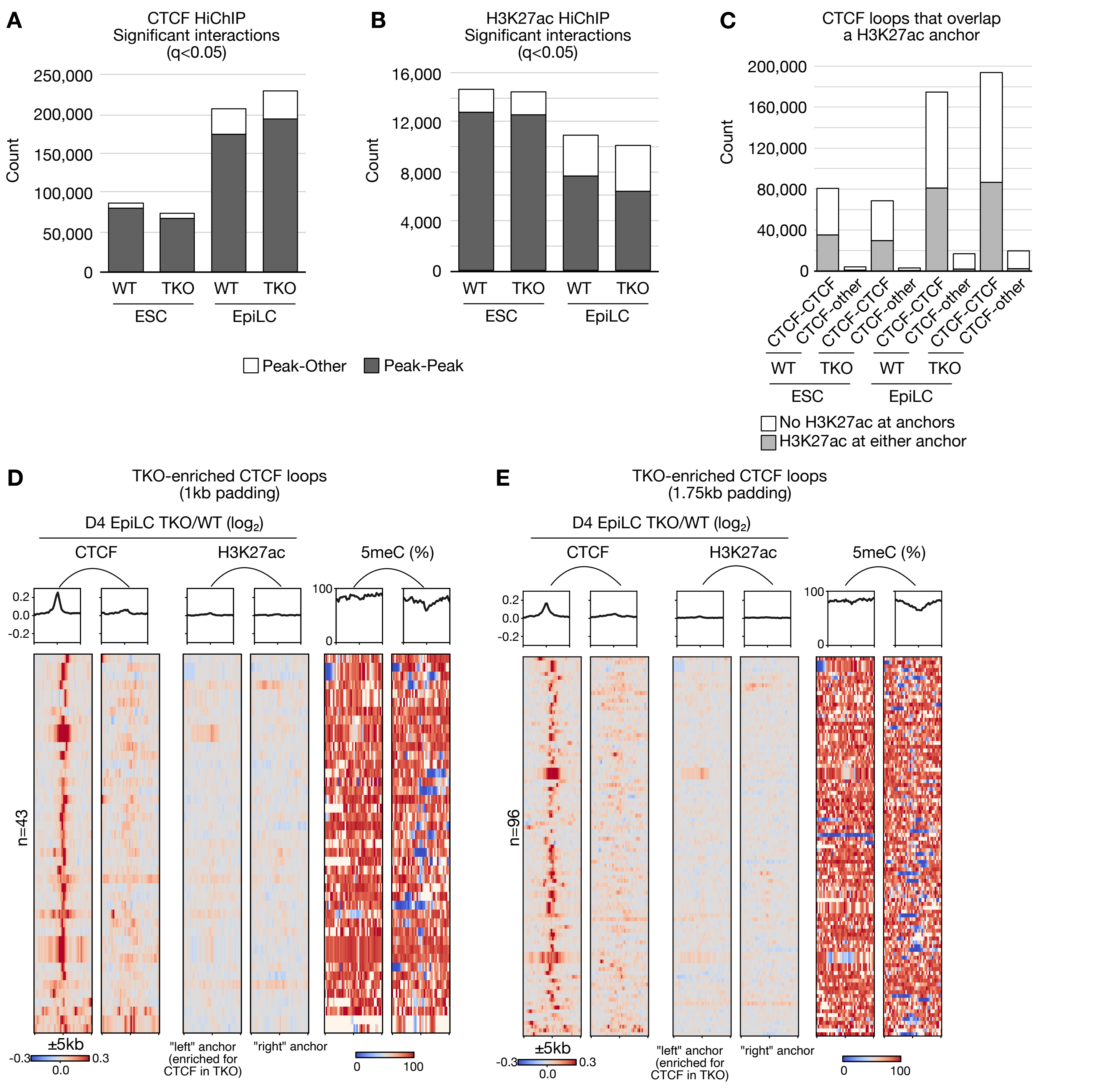

Supplementary Figure S5 – Integrative analysis of CTCF and H3K27ac HiChIP data.

A. Bar plot showing the number of CTCF-CTCF "peak-to-peak" and CTCF-other "peak-other" 3D interactions.

B. Bar plot showing the number of H3K27ac-H3K27ac "peak-to-peak" and H3K27ac-other "peak-other" 3D interactions.

C. Bar plot showing the number of CTCF-CTCF and CTCF-other 3D interactions that overlap an H3K27ac peak at either anchor.

D. Average profile and heatmaps showing the log_2 fold-change in CTCF and H3K27ac enrichment at TKO EpiLC-specific CTCF-CTCF loops. Both anchors of each loop are represented, and the anchor showing TKO-specific CTCF binding is sorted on the left. 5meC levels in WT are included. TKO-specific loops were identified by counting the number of CTCF-CTCF contacts over CTCF peaks +/- 1kb.

E. As in **D**, but considering CTCF-CTCF contacts over CTCF peaks +/- 1.75kb.

Supplementary Figure S6

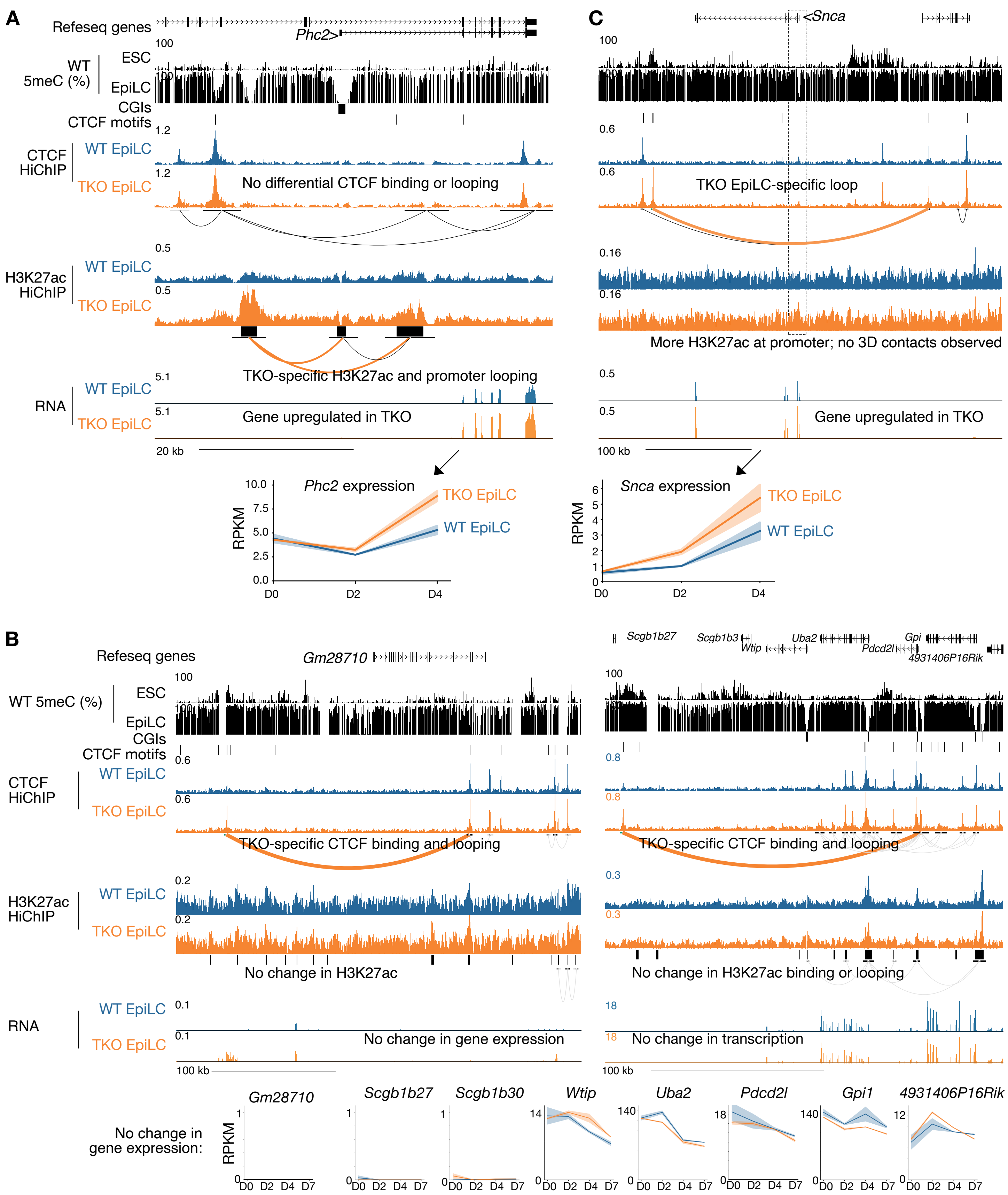

Supplementary Figure S6 – Examples of differentially regulated genes in TKO EpiLCs that did not meet criteria for further analysis.
A. UCSC Genome Browser screenshot of the *Phc2* locus, showing no detected differential CTCF looping (CTCF HiChIP) despite a TKO-specific increase in H3K27ac interactions and gene expression. Statistically enriched CTCF loops in TKO EpiLCs are shown in orange. Gray loops represent significant interactions that are consistent across WT and TKO EpiLCs. Enhancers are shown below H3K27ac tracks. Gene expression levels in ESCs, day 2 (D2) and 4 EpiLCs are shown below as a line plot, where the line represents the mean and the shaded area the standard deviation (n=2).
B. Screenshot of the *Gm28710* and *Scgb1* loci showing TKO-specific CTCF looping (orange) with no change in underlying H3K27ac enrichment or looping or transcription. Tracks are shown as in **A**.
C. Screenshot of the *Snca* locus, showing TKO-specific CTCF looping and transcriptional upregulation in the absence of H3K27ac enrichment or looping. Tracks are shown as in **A.**

Supplementary Figure S7

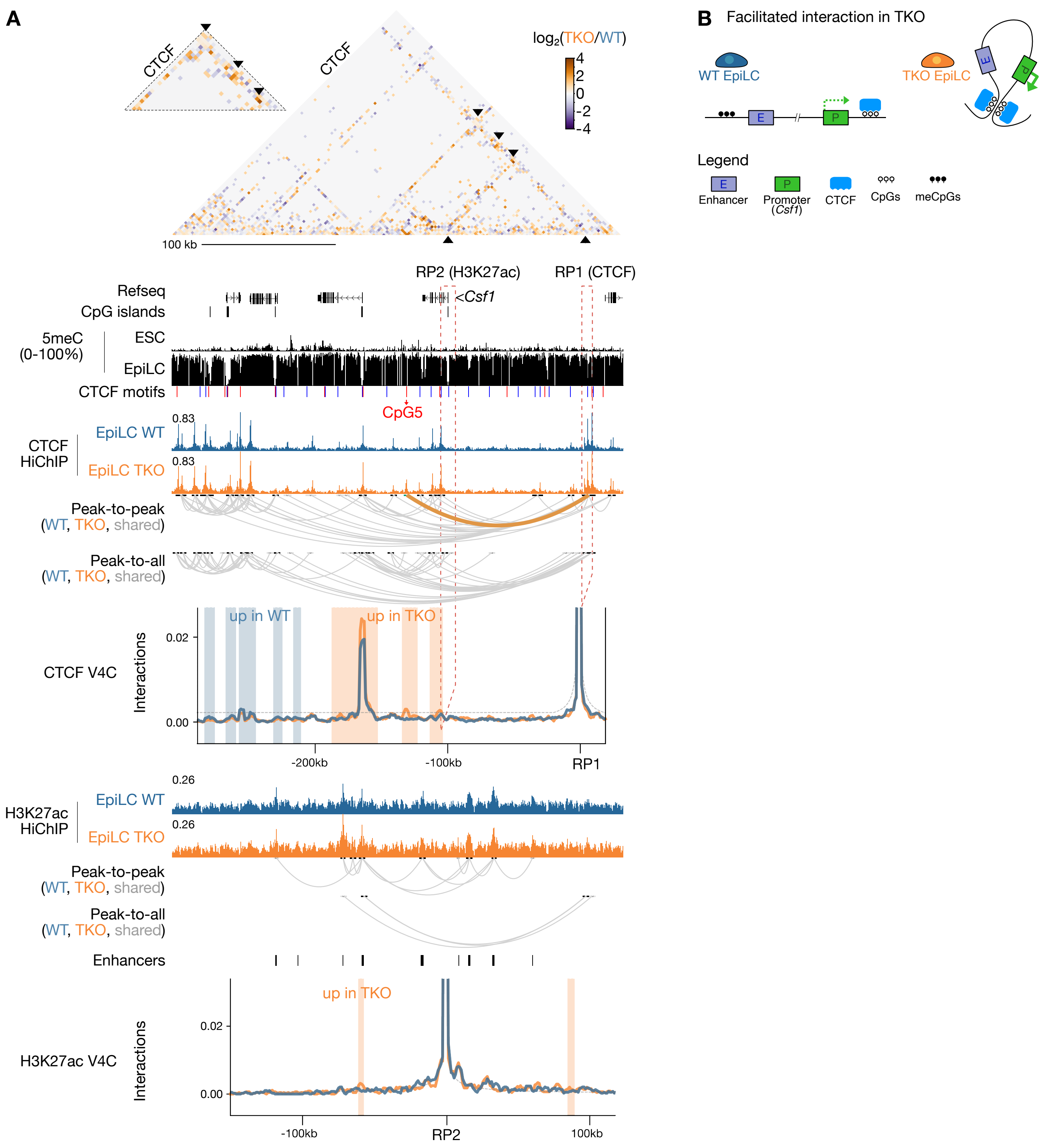

Supplementary Figure S7 – 5meC-sensitive CTCF looping is associated with increased promoter-enhancer contacts and gene expression at the *Csf1* locus in TKO EpiLCs.
A. Contact matrix (top), genome browser screenshot (middle) and virtual 4C (bottom) plots of the *Csf1* locus. Top: differential CTCF contacts between WT and TKO EpiLCs are displayed, where each pixel represents a 1 kb bin. The inset magnifies the TKO-enriched CTCF contacts. Reference points (RP) 1 (CTCF) and 2 (H3K27ac) for the virtual 4C plots (bottom) are indicated by a dashed rectangle. Middle: browser screenshot showing CTCF and H3K27ac enrichment levels and 3D interactions (peak-to-peak and peak-to-all). TKO-specific loops are shown in orange, and gray loops represent significant interactions that are consistent across WT and TKO EpiLCs. Refseq genes, CpG islands, CpG methylation levels, CTCF motifs (positive strand in red, negative strand in blue) are included. The CTCF motif with a CpG at position 5 that underlies a putative 5meC-sensitive CTCF peak is highlighted. Bottom: Virtual 4C plots of reference points 1 (CTCF) and 2 (H3K27ac) showing interaction frequencies between the reference point and adjacent area. The background model is shown as a dotted gray line. Statistically enriched contacts (chi-squared test, alpha<0.25) are highlighted in blue (high in WT EpiLC) or orange (high in TKO EpiLC). Coordinates: chr3:107,554,568-107,890,568.
B. Schema depicting the hypothesized facilitated enhancer-promoter interactions and increased gene expression in TKO EpiLCs.

Supplementary Figure S8

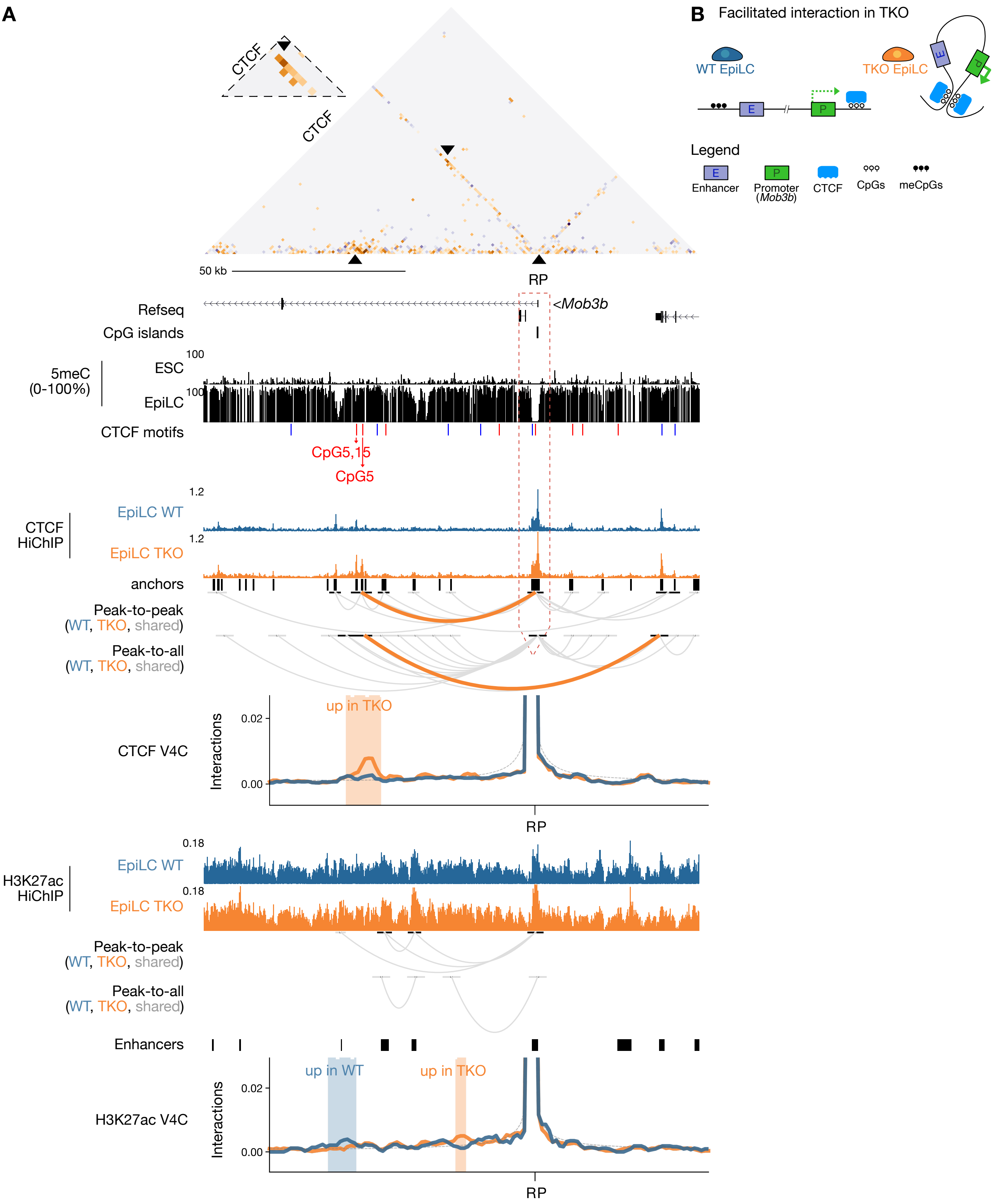

Supplementary Figure S8 – 5meC-sensitive CTCF looping is associated with increased promoter-enhancer contacts and gene expression at the *Mob3b* locus.
A. Contact matrix (top), genome browser screenshot (middle) and virtual 4C (bottom) plots of the *Mob3b* locus as in **Supplementary Figure S7**. Coordinates: chr4:35,061,303-35,203,887.
B. Schema depicting the hypothesized facilitated enhancer-promoter interactions and increased gene expression in TKO EpiLCs.

Supplementary Figure S9

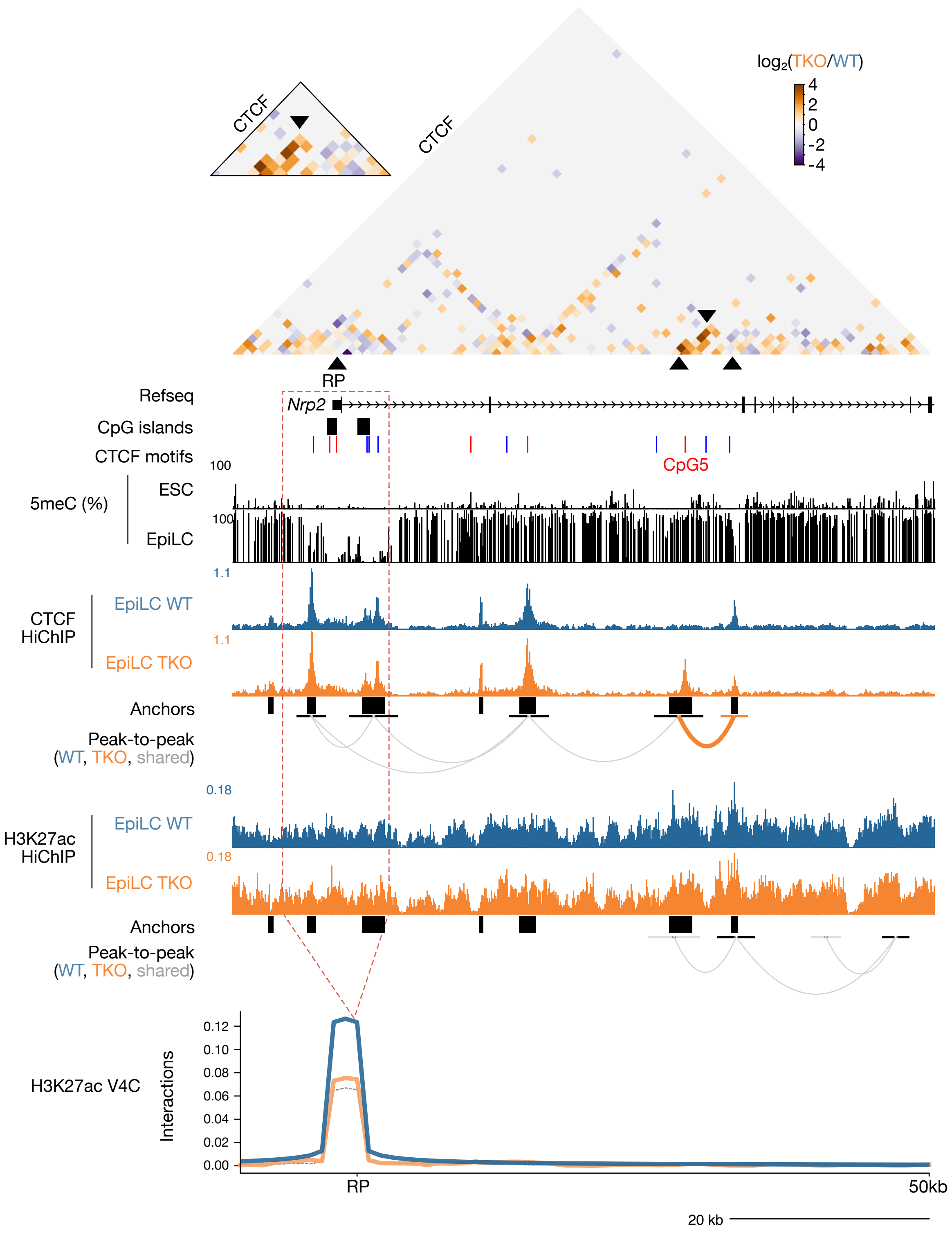

Supplementary Figure S9 – 5meC-sensitive CTCF looping at the *Nrp2* locus.
Contact matrix (top), genome browser screenshot (middle) and virtual 4C (bottom) plots of the *Nrp2* locus as in **Supplementary Figure S7**. Note the lack of differential H3K27ac interactions between the promoter and adjacent sequences. No peak-to-all interactions were identified. Coordinates: chr1:62,693,316-62,763,316.

Supplementary Figure S10

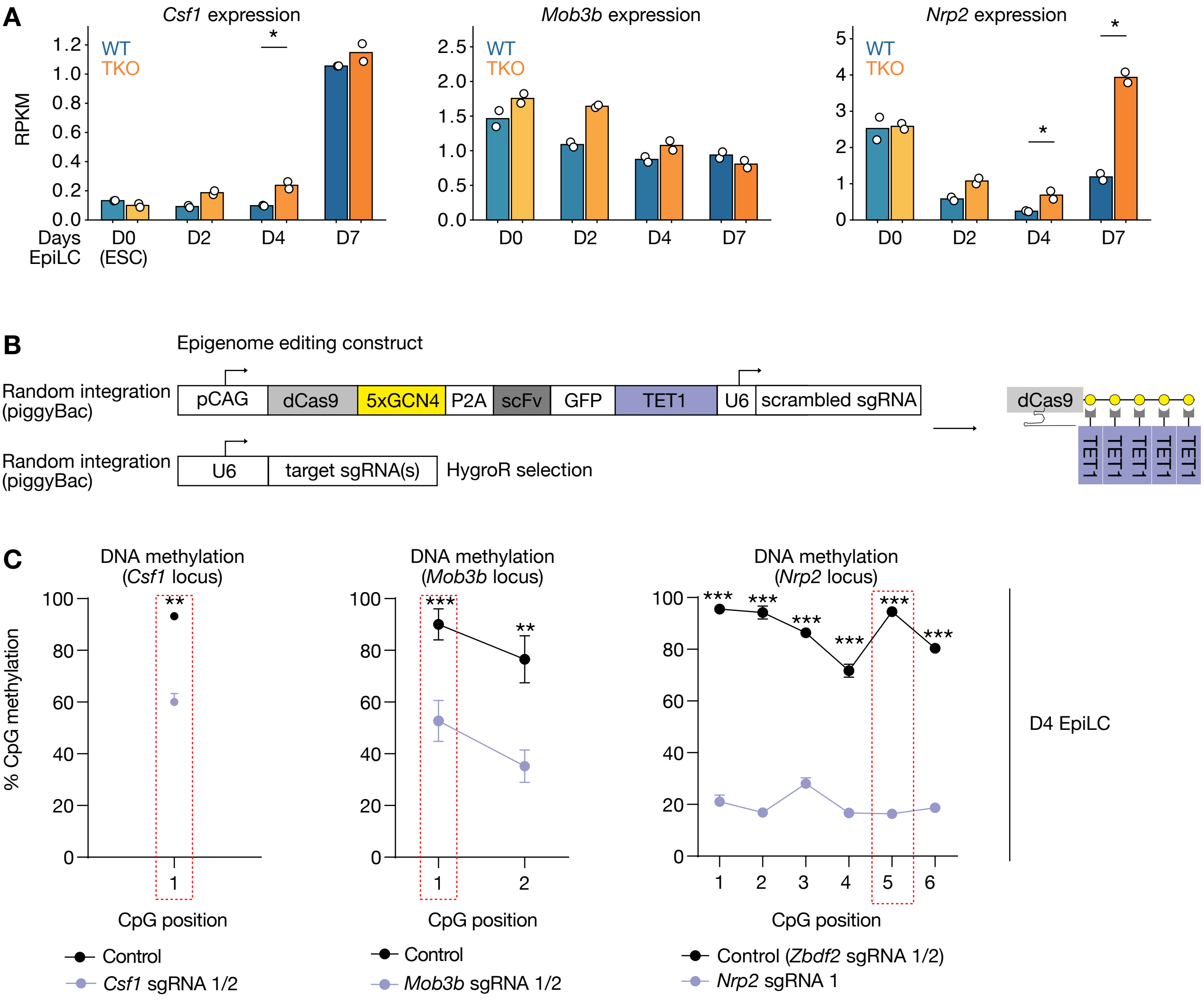

Supplementary Figure S10 – Site-directed cytosine demethylation at candidate 5meC-sensitive CTCF loops.
A. Heatmap showing expression levels (RPKM) of genes near 5meC-sensitive CTCF loops over a 7-day EpiLC differentiation time course. Time points where genes are scored as differentially expressed (linear modeling using Limma, t-test, $\log_2$ fold-change>1, adjusted p value<0.05) are indicated by an asterisk.
B. Epigenome editing construct. A constitutive CAG promoter drives expression of a catalytically inactive Cas9 (dCas9) fused to 5xGCN4 epitopes (SunTag, yellow circles), a self-cleavable peptide (P2A), and a single chain variable fragment (scFv) that recognizes GCN4 epitopes fused to GFP and the human TET1 catalytic domain. This construct also contains a scrambled sgRNA sequence under control of a U6 promoter. A separate construct containing U6 promoter driving expression of targeted sgRNAs was transfected for experiments and individual loci. Both constructs were stably inserted in the genome by piggyBac transposition; the dCas9-SunTag/TET1 construct was selected by sorting GFP expressing cells, and the sgRNA construct by hygromycin selection.
C. Bisulfite-pyrosequencing results of epigenome edited D4 EpiLCs. The CpG corresponding to CpG5 in the canonical CTCF binding motif are highlighted by red boxes. Data are shown as mean ± standard error for three replicates. p-values were calculated by two-tailed paired t-test: *p<0.05, **p<0.01, **p<0.001, ***p<0.0001.

Supplementary Figure S11

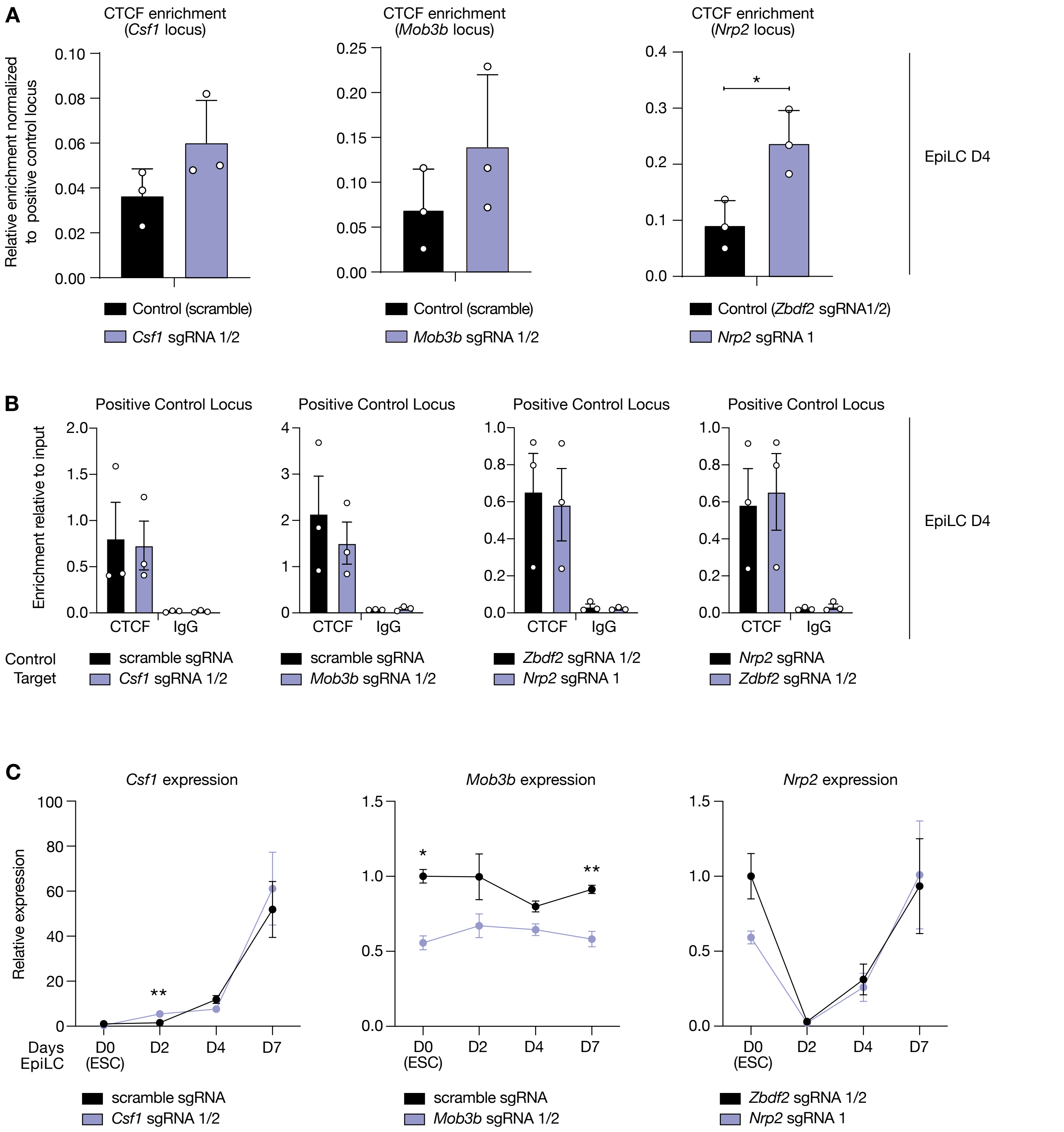

Supplementary Figure S11 – Site-specific cytosine demethylation results in increased CTCF recruitment but modest changes in nearby gene expression levels.
A. CTCF ChIP results with targeted cytosine demethylation in D4 EpiLCs. Data are shown as mean ± standard error for three replicates represented by unfilled circles.
B. CTCF and IgG (background) ChIP at positive control locus (chr1:63,181,149-63,181,244) relative to input DNA in D4 EpiLCs. Data are shown as mean ± standard error for three replicates represented by unfilled circles.
C. RT-qPCR results of the same cells as in **A** over a time course of 7 days of EpiLC differentiation. Expression of each replicate was normalized to two housekeeping genes (*Rrm2* & *Rplp0*), and then to WT ESCs. Data are shown as mean ± standard error for three replicates. Data are shown as mean ± standard error for three replicates. p-values were calculated by two-tailed paired t-test assuming unequal variance: *p<0.05, **p<0.01, **p<0.001.

Supplementary Figure S12

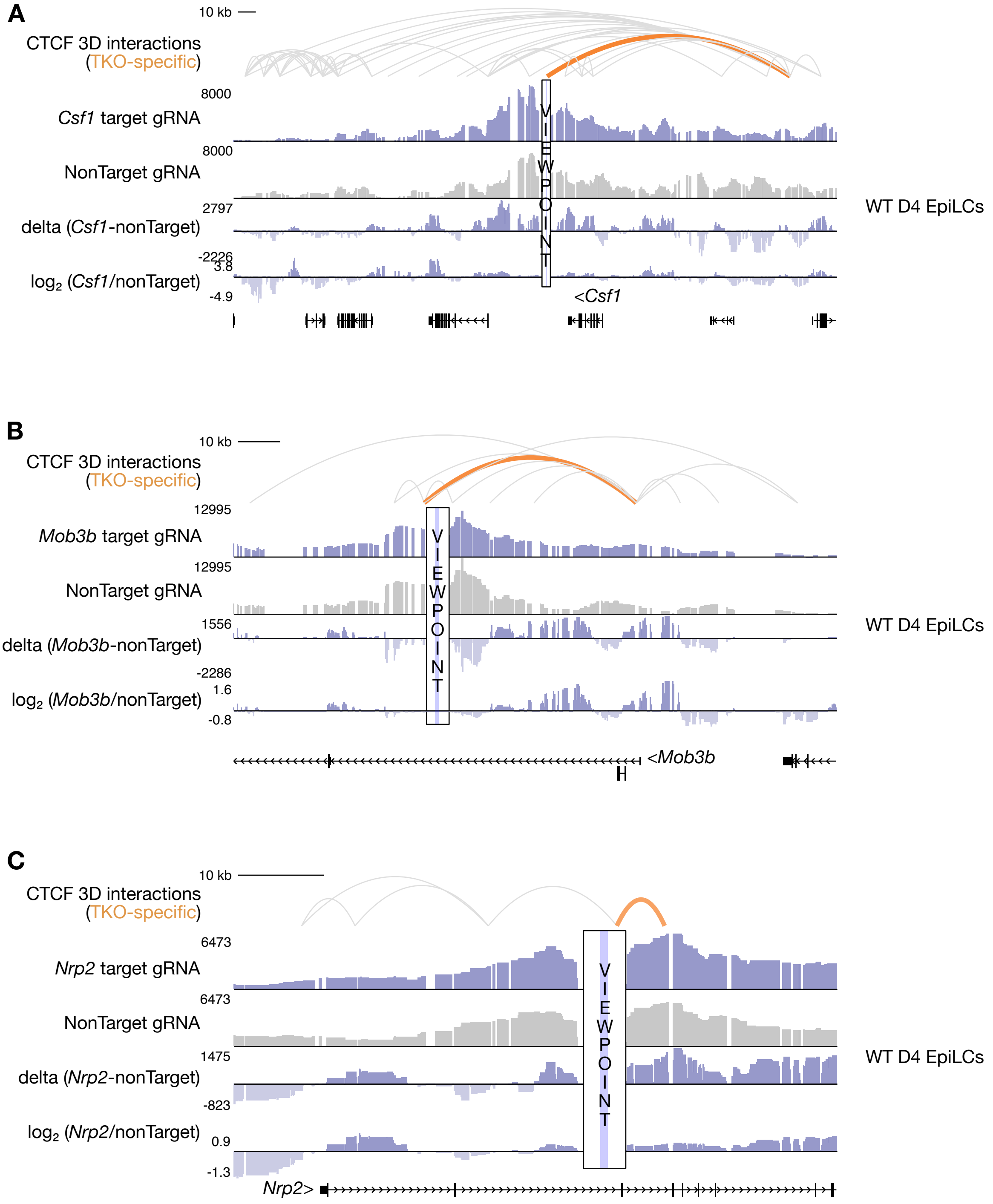

Supplementary Figure S12 – Site-specific cytosine demethylation results in the recreation of a TKO-specific CTCF loop in EpiLCs at some loci.
A. UCSC Genome Browser screenshot of the *Csf1* locus showing the TKO-specific CTCF peak (the 4C-seq viewpoint) and loop (HiChIP, orange). Shared loops (gray). TET1-mediated epigenome edited Day 4 EpiLC 4C-seq is shown for targeted (purple) and non-targeted (grey) samples. The delta and $\log_2$ fold-change between target and non-target 4C-seq samples are included, where positive values indicate enrichment in the epigenome edited cells. Coordinates: chr3:107,554,568-107,890,568
B. The *Mob3b* locus as in **A**. Coordinates: chr4:35,061,303-35,203,887.
C. The *Nrp2* locus as in **A**. Coordinates: chr1:62,693,316-62,763,316

Supplementary Figure S13

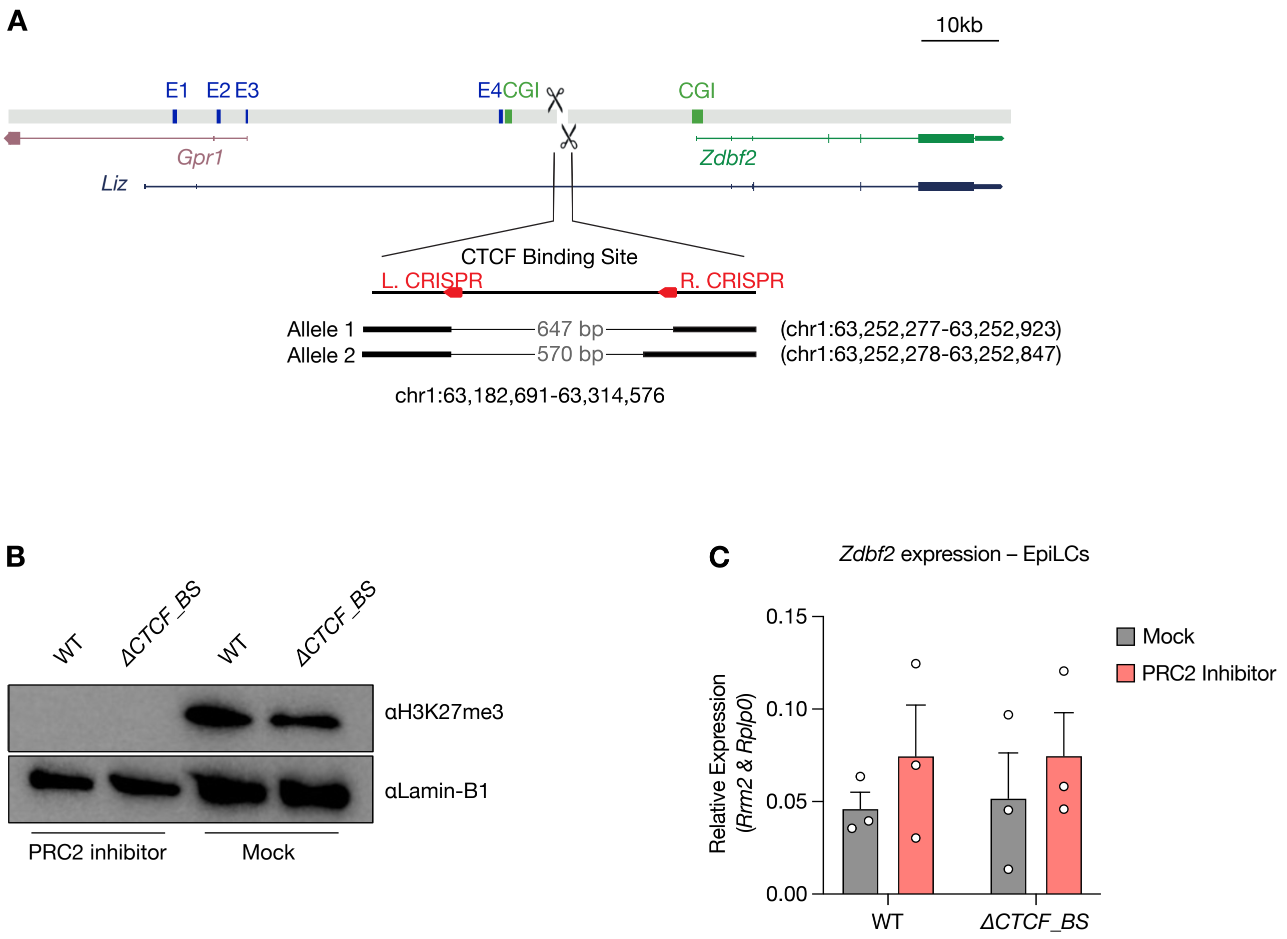

Supplementary Figure S13 – Genetic ablation of a 5meC-sensitive CTCF binding site.
A. Schema depicting target Cas9 target sites (red), previously defined enhancers (E1-4, blue) and CpG islands (CGI, green) at the *Zdbf2* locus. The entire window spans chr1:63,182,691-63,314,576 and the length and coordinates of the deletions are indicated.
B. Western blot showing H3K27me3 levels in WT and CTCF binding site-deleted (*ΔCTCF_BS*) ESCs grown in 2i+vitC. Cells were treated with UNC1999 (PRC2 inhibitor) or UNC2400 (mock). Lamin-B1 was used as a loading control.
C. Relative *Zdbf2* expression levels in EpiLCs treated with UNC1999 or UNC2400. Expression levels were normalized to housekeeping genes *Rrm2* and *Rplp0*. Data are shown as mean ± standard error for three replicates, and individual replicates. No statistically significant differences were measured.
